# Relating Approach-to-Target and Detection Tasks in Animal Psychoacoustics

**DOI:** 10.1037/bne0000143

**Published:** 2016-05-19

**Authors:** Joseph Sollini, Ana Alves-Pinto, Christian J. Sumner

**Affiliations:** 1 Medical Research Council Institute of Hearing Research, Nottingham, United Kingdom

**Keywords:** psychoacoustics, animals, signal detection, localization, behavior

## Abstract

Psychophysical experiments seek to measure the limits of perception. While straightforward in humans, in animals they are time consuming. Choosing an appropriate task and interpreting measurements can be challenging. We investigated the localization of high-frequency auditory signals in noise using an “approach-to-target” task in ferrets, how task performance should be interpreted in terms of perception, and how the measurements relate to other types of tasks. To establish their general ability to localize, animals were first trained to discriminate broadband noise from 12 locations. Subsequently we tested their ability to discriminate between band-limited targets at 2 or 3 more widely spaced locations, in a continuous background noise. The ability to discriminate between 3 possible locations (−90°, 0°, 90°) of a 10-kHz pure tone decreased gradually over a wide range (>30 dB) of signal-to-noise ratios (SNRs). Location discrimination ability was better for wide band noise targets (0.5 and 2 octave). These results were consistent with localization ability limiting performance for pure tones. Discrimination of pure tones at 2 locations (−90/left, 90/right) was robust at positive SNRs, yielding psychometric functions which fell steeply at negative SNRs. Thresholds for discrimination were similar to previous tone-in-noise thresholds measured in ferrets using a yes/no task. Thus, using an approach-to-target task, sound “localization” in noise can reflect detectability or the ability to localize, depending on the stimulus configuration. Signal-detection-theory-based models were able to account for the results when discriminating between pure tones from 2- and 3-source locations.

The aim of perceptual psychophysical testing is to measure the limits of perception. This can often be difficult in animal models where weeks or months are required to train the animals (Birch, Warfield, Ruben, & Mikaelian, 1968; Ehret, 1974; Heffner & Heffner, 1980; Klink, Bendig, & Klump, 2006; Prosen, Dore, & May, 2003; Radziwon et al., 2009; Zheng, Johnson, & Erway, 1999), and in some instances some tasks are not learned at all (Burdick, 1979; Goltstein, Coffey, Roelfsema, & Pennartz, 2013). The important challenges are to choose and refine the appropriate task for a given experimental question in a given species, and then to interpret that data correctly.

Distinct types of operant conditioning tasks have evolved for psychophysical testing. These require the animal to choose between several discrete actions. In a typical single-interval two-alternative forced-choice (1I2AFC) task an animal initiates a trial (with a lever press, nose poke, or by licking a spout), which produces a sensory stimulus. They must then choose between two alternative responses. In a positive reinforcement task they will receive a reward (usually food or water) for choosing the correct response (contemporary examples: Alves-Pinto, Sollini, & Sumner, 2012; Otazu, Tai, Yang, & Zador, 2009; Walker, Schnupp, Hart-Schnupp, King, & Bizley, 2009). Another commonly used type of task is the go/no-go (contemporary examples: Brosch, Oshurkova, Bucks, & Scheich, 2006; Hienz, Stiles, & May, 1998; Klink et al., 2006; Prosen, Bath, Vetter, & May, 2000). Here, the subject must remain inactive, waiting for a stimulus (detection) or change in a repeated stimulus (discrimination), whereupon they must respond within a certain time.

These tasks can be criticized in several respects, the first relates to signal detection theory (SDT, MacMillan & Creelman, 2005). According to SDT, a psychophysical task can be formalized as a process in which noisy sensory input (usually a single variable) is compared to a criterion value to yield a decision. In both 1I2AFC and go/no-go tasks, the decision criterion is set by prior experience in the task and remembered. Optimal setting of the criterion will maximize the percent correct (performance) and minimize any tendency to preferentially respond one way independent of the stimulus (bias). Although bias can be reasonably accounted for by SDT analysis methods (Blough, 2001), researchers in human psychophysics often use task design to alleviate it (MacMillan & Creelman, 2005). For example, by presenting two stimuli sequentially (two-interval task) and in random order where the listener must choose the interval with the “target” stimulus (2I2AFC). This has the advantage of decoupling response bias from the stimulus dimension because any bias will now be toward a given interval rather than a particular stimulus (presentation order is independent of the stimulus). As the two sequentially presented intervals can be compared, this task also reduces memory demands (in humans at least). Unfortunately two-interval tasks are difficult to train in animals, unless the first interval is a “reference” (Walker et al., 2009). This may reduce memory demands but by not randomizing the stimulus order response bias is once again coupled to the stimulus dimension.

A second criticism is that 1I2AFC and go/no-go tasks require subjects to associate an arbitrary relationship between a stimulus (e.g., a sound) and a response choice (“yes/no” or “go/no-go”). Classical studies into animal behavior have shown that the rate of acquisition depends on the type of association that must be learned (reviewed by Burdick, 1979). Harrison and colleagues demonstrated that when an auditory stimulus (the speaker) and the response (lever) were in close proximity animals naturally responded correctly, even on the first trial (Harrison, 1984, 1992; Neill & Harrison, 1987). Harrison (1992) emphasized the importance of considering an animal’s natural behavior when deciding on an appropriate task. In a natural environment, sounds come from objects and have spatial locations. The relationship between a sound and the response is unlikely to be arbitrary.

Here, we examine whether an approach-to-target task (e.g., Nodal, Bajo, Parsons, Schnupp, & King, 2008) can be used to measure auditory detection thresholds. This task takes natural advantage of the colocation of the stimulus and response. Second, the stimulus feature determining the response (location) is different from the feature of interest (sound level or signal-to-noise ratio [SNR]). Thus, like a 2I2AFC task, any response bias does not manifest as a shift in decision criterion along the stimulus dimension. Additionally, if the same basic responses are generalizable, it would allow both types of measurement in individual animals without the need to train on two separate tasks (Kavanagh & Kelly, 1988).

This comes with some considerations. Sound detectability and localizability are interrelated: As the probability of detecting a sound is reduced, localization errors increase (Good & Gilkey, 1996; Jacobsen, 1976; Lorenzi, Gatehouse, & Lever, 1999b; Sabin, Macpherson, & Middlebrooks, 2005). For humans localizing sounds in noise, performance begins to become affected between 0 and 6 dB SNR (Good & Gilkey, 1996; Lorenzi et al., 1999b), whereas detection thresholds in noise are much lower than this (Lorenzi et al., 1999b). This would suggest using a sound localization task to measure detection thresholds would result in elevated “detection” thresholds. These studies, however, were conducted using the full 360° of the azimuthal plane. Reducing the number of response locations and increasing the angle between sound sources improves localization ability (Hartmann & Constan, 2002).

The experiments reported here examine constraints on the localization of signals in background noise, with the aim of determining whether and when performance in an approach-to-target task is consistent with psychophysical detection thresholds.

## Materials and Method

### Subjects

Subjects were three male and one female pigmented ferrets (*Mustela putorius*). Animals’ access to water was regulated, and water was used as positive reinforcement during training where most of their daily water was received. Supplemental water was given at the end of the day, mixed with ground ferret food and a supplement (Cimicat, Petlife International Ltd., U.K.). Animals performed two to three behavioral sessions a day for 10–14 days and were then rested and provided with water ad libitum for a minimum of 3 days. All ferrets were weighed daily and their health monitored continuously. Behavioral testing was stopped if an animals’ weight dropped below 20% their preregulation weight or if there were any other health concerns. Animals were housed individually with environmental enrichment in their cages and received daily social activity with other ferrets. All procedures were carried out under license from the U.K. Home Office.

### Apparatus

**Figure 1 fig1:**
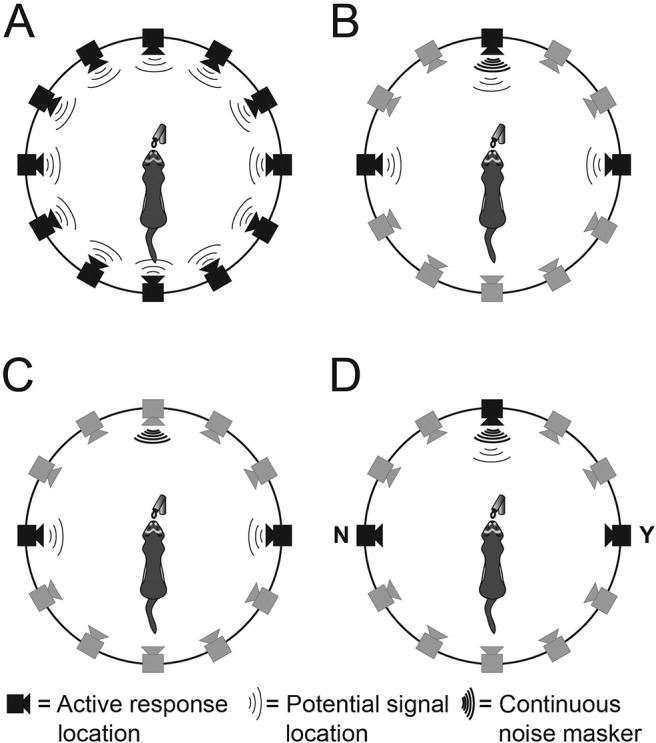
Schematics of the behavioral arena layout and stimulus locations. (A) Twelve-location localization paradigm. (B) Three-location discrimination task. (C) Two-location discrimination task. Animals were trained to lick at a central water spout to trigger a sound from anyone of the potential signal locations. A response could then be given at any one of the active response locations. (D) Yes/no task. All sounds came from 0° and responses were made to the left (N) and right (Y). In the discrimination tasks (B, C, and D), a continuous noise masker was presented from straight ahead.

Figure 1 shows the behavioral apparatus. Animals were tested in a circular wire-caged arena with a polyvinyl chloride floor measuring 150 cm in diameter, inside a sound-attenuated room, as described in other studies (Alves-Pinto et al., 2012). The arena was surrounded with an acoustically transparent net fabric, behind which were 12 modules separated by 30° along the azimuthal plane (covering 360°). Each module had a speaker (Visatron FX10; 70–22 kHz) for sound delivery and a water spout for water delivery. Licks were detected using an infrared beam and detector housed within each spout. In the center of the arena floor was a raised platform with a low fence on three sides, two side posts containing an infrared light-emitting diode and detector, and a water spout with a wire nose cone. A trial was triggered when both the infrared beam between the fence posts was broken and a lick was registered at the infrared sensor in the center spout. Behavioral sessions were controlled by custom software run on a PC. Licks were detected and sound presented via an analog/digital/analog (ADA) converter (MOTU 24 I/O). Water pumps were controlled via a custom made USB controller, which delivered ∼300 μl (in 30 μ drops) for each correct response.[Fig-anchor fig1]

### Stimuli

Stimuli were broadband noise, band-pass noise and 10-kHz pure tones, ranging in duration from 40 ms to 3 s and sampled at 96 kHz. The broadband noise stimuli were generated from a 30-s wide-band frozen noise. Two bandpass noise targets centered at 10 kHz, with half-octave and two-octave bandwidths were generated by filtering the broadband noise using a Butterworth filter (108 dB per octave roll off). Noise target stimuli were generated from broadband and bandpass noises by randomly selecting a section of the correct duration on each trial. Target stimuli varied in duration, and all were gated with a 10-ms cos^2^ ramp. The continuous broadband masker (experiments two and three) was made by continuously looping the broadband noise. The sound pressure level (SPL; root mean square) was measured with a 0.5-in. B&K 4165 condenser microphone (Bruel and Kjaer, Copenhagen, Denmark), pointing upward and placed at a position where the ferret’s head was when a trial was triggered.

### Training

Ferrets were first “shaped” to approach and lick the center spout in the absence of sound. The ferret was rewarded every time it licked the center spout, after the reward was given no more reward would be given until the ferret left the center spout and then returned. To encourage correct body position (the ferret entering the fence and facing the spout), reward was only delivered when increasingly closer approximations of good body and head position were achieved. This continued until reward was only given when ferrets repeatedly assumed the correct body position.

Once shaping was complete (generally three to four sessions), animals were trained to localize sound sources. Licking at the center spout triggered a trial, with a water reward. A 30-s broadband noise was presented from one of the 12 surrounding speakers, with a random delay from the start of the trial (0.5–2 s). Animals tended to instinctively approach the target location and were rewarded for correctly localizing a sound. If they did not answer correctly, no reward was given and the trial would be repeated with another noise burst from the same speaker at the same sound level. This continued until they answered correctly, whereupon a reward was delivered. Repeated trials were not included in any data analysis. The number of drops delivered at the center spout was systematically reduced (from 10 on every trial to one drop every 10 trials) across sessions at a rate dependant on performance. Then the reward at the peripheral spouts was adjusted to maximize the number of trials the ferret performed (the goal being >100 trials). Once a satisfactory reward balance had been met, signal durations were gradually reduced. The duration was reduced when performance reached and remained at >85% for a minimum of three behavioral sessions. Animals were considered trained when they could localize 1-s noise bursts from 12-speaker locations at >85% correct.

### Twelve-Speaker Localization

In the 12-speaker localization task, a broadband noise (randomly roved between 65 and 75 dB SPL) was presented on each trial from one of the 12 surrounding speaker modules (Figure 1A), in pseudorandom order. Correct answers were rewarded and incorrect trials were repeated as during training. Sound localization ability was measured at different signal durations, which were systematically reduced from 1 s down to either 0.2 s or 0.04 s. At each duration, performance was measured over two to three sessions of stable performance.

### Three-Location Discrimination

In the three location paradigm, the target sound was either a 0.5-s pure tone, half-octave, or two-octave band-pass noise (all centered at 10 kHz). Targets could be presented from one of three surrounding speaker locations: left (−90°), straight ahead (0°), or right (90°; Figure 1B). As before, the animal would then respond by licking at one of these modules and answers at the target location were rewarded. All animals had previously been trained on the 12-location speaker localization task. Initially, pure tone targets were used with long durations (10–30 s) and a randomized sound level across trials (60–80 dB SPL). A broadband masker was then introduced, and presented continuously from directly in front of the central platform (0°) at 34, ferrets: *F*(1, 2), or 46 dB SPL: *F* (3, 4). Target durations were then systematically reduced, as before. Animals were considered trained when overall performance of >85% correct was gained for a 0.5-s duration signal.

During testing the signal level was then systematically reduced using the method of limits. The signal level started at a high SNR (>20 dB) to elicit good performance (>85% correct). Comparable performance (within 5% of each other) on two sessions was required before the signal level was reduced (by 5 dB). Once performance had fallen to ∼40% correct, the behavioral block was ended. This process was repeated for all three signal bandwidths.

### Two-Location (Left/Right) Discrimination

In the two-location discrimination paradigm (Figure 1C) signals were presented from one of two surrounding speaker locations: left (−90°) or right (90°). A continuous broadband masker was presented from straight ahead (0°) as in the three-location discrimination. Animals had already been trained with 12 and then three-speaker locations. To make the transition from a three- to a two-location (left/right) discrimination task, animals were first acclimatized localizing 0.5-s noise bursts for four sessions. After this the stimulus was then changed to a 0.5-ms duration 10-kHz pure tone. Animals were tested using this stimulus until performance had reached >85% correct on two consecutive sessions. Generally, only two sessions were required to reach this criterion. In all other respects (reward, stimulus, and reward timing, etc.), the conditions were as described for the previous experiments.

The ability of ferrets to discriminate tones at ±90° (left/right) was measured at different levels in the presence of a continuous noise masker, to yield psychometric functions and thresholds. Two methods were compared: the method of limits and the method of constant stimuli. The method of limits procedure was the same as that described in the three-location paradigm. For the method of constant stimuli, five to seven levels were selected for each session on the basis of pilot measurements in each individual ferret. One sound level was chosen to deliver high levels of performance. Other sound levels were chosen, usually at 5-dB intervals, to span the steepest part of the psychometric function. Performance using these levels was then collected for at least 20 sessions (>2,000 trials).

### Yes/No Detection Task

Thresholds derived from the two-location discrimination task were compared quantitatively with baseline tone-in-noise detection thresholds measured using a single interval, two-alternative choice task (hereafter referred to as the “yes/no” or detection task; Figure 1D). This task has been used in numerous earlier studies in ferrets (Hine, Martin, & Moore, 1994; Kelly, Kavanagh, & Dalton, 1986; Neff, Diamond, & Casseday, 1975). These data were collected using the same behavioral apparatus and have been published previously (Alves-Pinto et al., 2012).

Briefly, ferrets initiated trials by licking the central spout, in which there was a 50% chance of a 10-kHz pure tone being played from the 0° speaker (signal trials). On the other half of trials no sound was presented (no-signal trials), but on all trials a light-emitting diode was illuminated from the location of the speaker. Signal trials were rewarded when responses were made at the spout located at 90° (“yes” response), while no-signal trials were rewarded at the −90° spout (“no” response). Incorrect responses were not rewarded, and the subsequently triggered trial was identical to the previous, as for the localization tasks. Signal and masker levels were comparable (35 or 48 dB SPL) with those used for the sound localization tasks, and psychometric functions were collected using the method of constant stimuli and the method of limits. Here, these data are reanalyzed using identical methods to the left/right discrimination data.

### Data Analysis

The data were analyzed by combining all trials for a given stimulus condition across behavioral sessions. Performance for 12- and three-location tasks was measured as simple percent correct for each stimulus condition. For two-location discrimination and yes/no detection tasks, we used standard signal SDT methods (MacMillan & Creelman, 2005) to compute PC_max_, a bias free measure of the discrimination between the two-alternative choices in each task (left/right and yes/no). For the yes/no task, PC_max_ at a given signal level is given by

$$PC_{\max}=\Phi \left[\frac{z(H)-Z(F)}{2}\right],$$ 1

where *z*(*H*) is the *z* score of the hit-rate for signals, *z*(*F*) is the *z* score of the false alarm rate for no-signal trials, and Φ[.] is the normal cumulative distribution function. To calculate false alarm rates for a given “signal level” in such a way that hit and false alarm rates were calculated for a similar number of trials, no-signal trials were randomly assigned a surrogate sound level from the range presented.

The left/right task also involves discriminating between two-alternative choices, and thus PC_max_ at a given signal level can be calculated as

$$PC_{\max}=\Phi \left[\frac{z(R_{L}|S_{L})-Z(R_{L}|S_{R})}{2}\right],$$ 2

where *z*(*R*_*L*_|*S*_*L*_) is the *z* score of responses to the left when the signal was presented from the left, and *z*(*R*_*L*_|*S*_*R*_) is the *z* score of responses to the left when the signal was presented from the right.

Thresholds were calculated for criterion values of 66% correct for three-location discrimination, and for a PC_max_ of 75% for the left/right discrimination and yes/no detection data, by linear interpolation between the two neighboring points. Confidence intervals for all measures were estimated by bootstrap resampling of the individual trials. The responses to each stimulus condition were resampled by drawing 100 responses from the complete set, with replacement. This resampled set of trials was then processed in the normal way. Resampling was performed 500 times for each condition. For any given measure (% correct, PC_max_, threshold) the mean and confidence intervals were determined from the numerical distribution of values.

### Experiment 1: Twelve-Source Sound Localization

**Figure 2 fig2:**
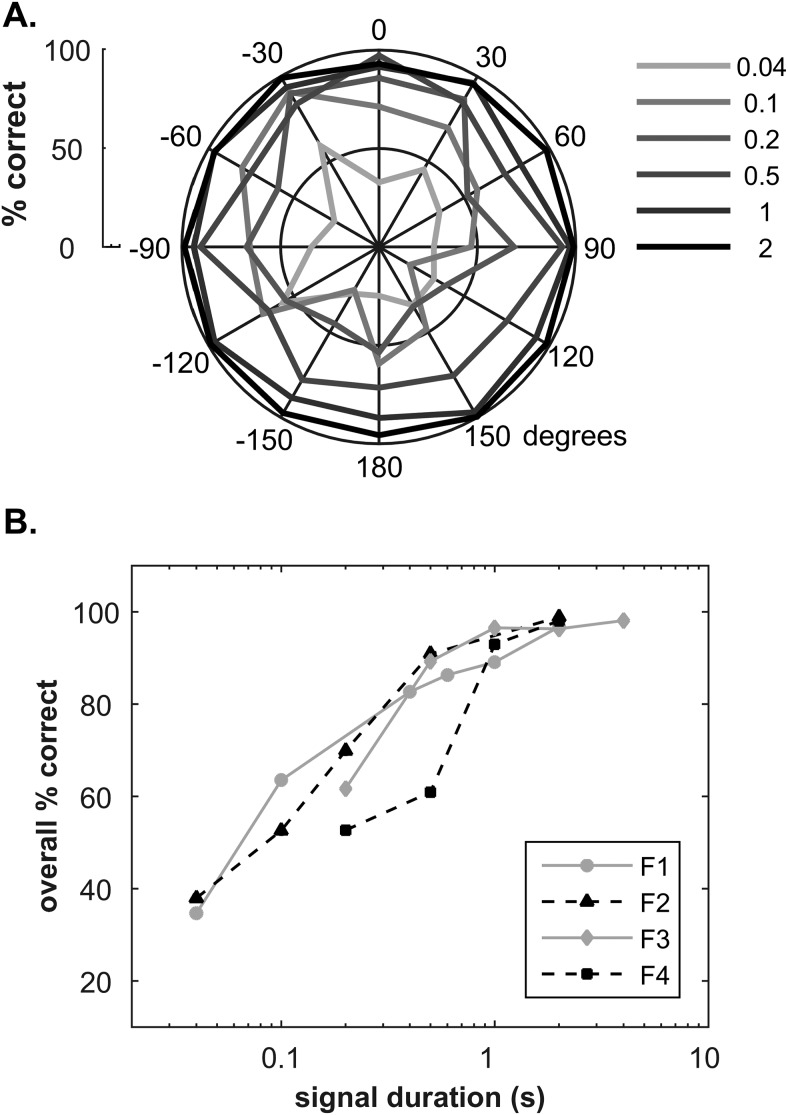
Localization of broadband noise bursts from 12 locations. (A) Percentage correct versus speaker location, averaged across F1–4, with duration of noise bursts indicated by the shade of gray. (B) Overall performance across speaker locations, for F1–4 individually.

Animals were first trained to perform the most general version of the approach-to-target localization task, following previous studies in ferrets (e.g., Irving, Moore, Liberman, & Sumner, 2011; Kacelnik, Nodal, Parsons, & King, 2006). Ferrets localized broadband noise bursts, which were presented from 12 possible locations at a randomized range of suprathreshold sound levels. Figure 2A shows the average percent correct at each speaker location for a range of target durations, averaged across animals (F1–F4). These data are also shown in Figure 2B, for each ferret individually but averaged across target location. In line with previous studies, ferrets were able to localize longer (≥1 s) duration noise bursts accurately and performance dropped at shorter durations, with frontal locations being more robust at shorter durations (≤0.2 s). A three-way analysis of variance (ANOVA) confirmed that there were significant differences with duration, *F*(8, 217) = 44.1, *p* < .0001, target location, *F*(11, 217) = 3.54, *p* < .001, and across animals, *F*(3, 217) = 4.03, *p* < .01.[Fig-anchor fig2]

These measurements established that ferrets had trained to localize broadband sounds from relatively arbitrary locations. While not essential for the measurements that followed, it ensured that ferrets initially learned to localize using binaural cues. It also served as an indication that ferrets could adapt to subsequent measurements rapidly from initial training on a more general sound localization task.

### Experiment 2: Three-Location Discrimination in Noise

The goal of Experiment 2 was to probe the limits of sound localization of bandlimited targets in background noise. Specifically, we examined the interaction of target bandwidth and the number and spread of locations across the frontal field with the aim of determining the constraints on localization performance for narrowband targets at low SNRs. Following testing on the 12-speaker localization task, ferrets were acclimatized on the three-location discrimination task. Band limited target signals centered at 10 kHz were presented at 0°, 90°, and −90°, and a range of signal levels, in the presence of a continuous broadband masker presented from directly in front of the animal (0°). Performance was measured at decreasing signal levels, using the method of limits (see Materials and Method). This was performed for 10-kHz pure tones, and half-octave and two-octave wide noise bursts.

## Results

**Figure 3 fig3:**
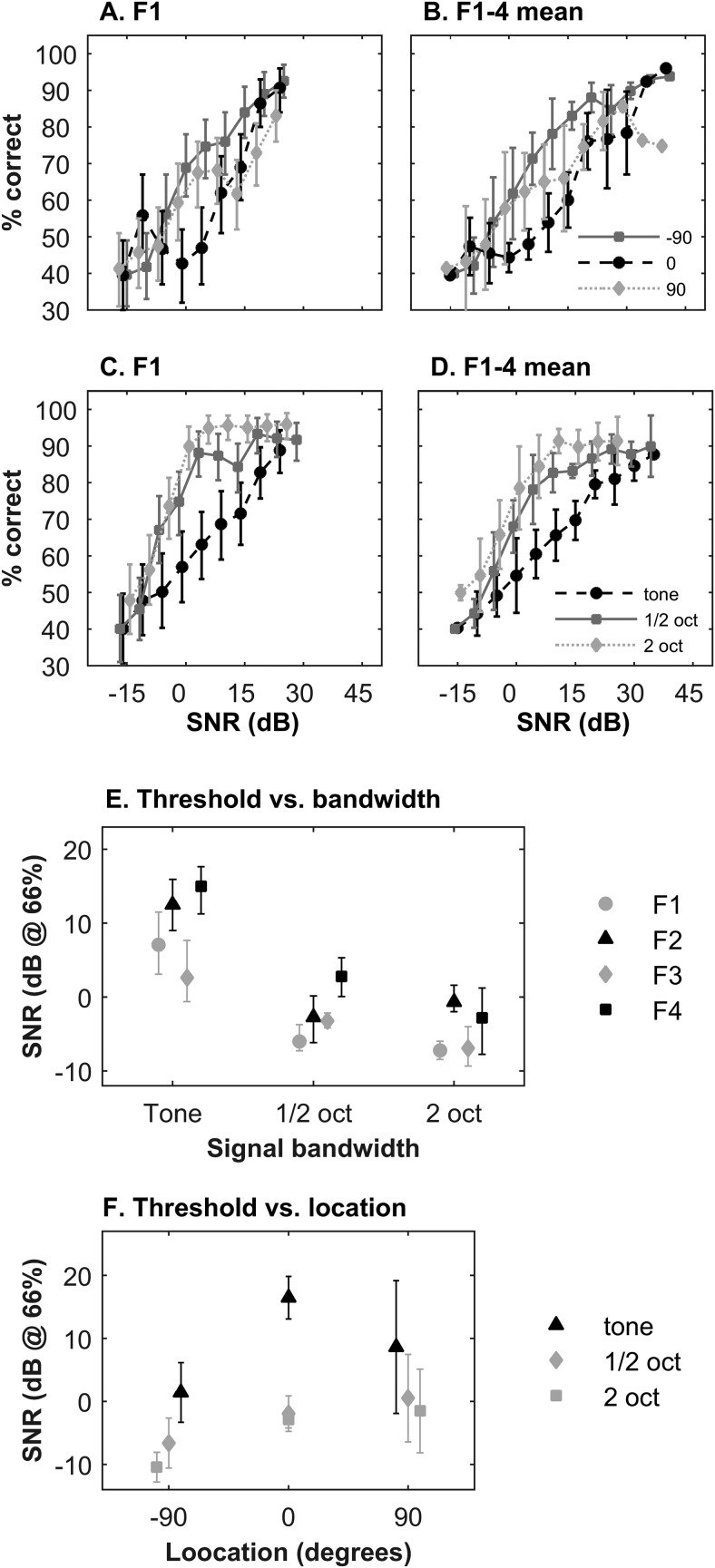
Three-location discrimination in background noise. (A) Performance as a function of signal-to-noise ratio (SNR) for each location of a 10-kHz pure tone target, for F1 only. (B) Average performance for F1–4 as a function of SNR for each location of 10-kHz pure tone. (C) Mean performance averaged across signal location, for F1 only, as a function of SNR and signal bandwidth. (D) Mean performance for F1–4, averaged across all signal locations, as a function of signal bandwidth. (E) Localization thresholds (66%) for each signal bandwidth. (F) Localization thresholds as a function of source location. Error bars in Parts A, C, and E indicate 95% confidence intervals. Error bars in Parts B, D, and F show standard deviation across animals. Note that in Part B, the two largest SNRs were only tested in one animal.

Figure 3A shows, for F1, the % correct at each target location when the targets were pure tones. Figure 3B shows these values averaged across animals. It is clear that locations are readily discriminable at high SNRs, and that performance decreased steadily with SNR. Lateral locations are easier to identify at all SNRs.[Fig-anchor fig3]

Figure 3C shows, for F1, the mean % correct across all target locations for pure tones, half-octave and two-octave noise bursts. Figure 3D shows the corresponding measurements averaged across animals. As the target bandwidths increase, performance increases and psychometric functions become steeper, with discrimination of noise-band location remaining largely constant at positive SNRs. A four-way ANOVA confirmed that there was a significant overall effect of SNR, *F*(10, 312) = 81.2, *p* < .0001, bandwidths, *F*(2, 312) = 85.7, *p* < .0001, and target location, *F*(2, 312) = 51.3, *p* < .0001, on performance, and also differences between ferrets, *F*(3, 312) = 16.6, *p* < .0001.

Figure 3E summarizes the effect of bandwidth on location discrimination for all animals (threshold at 66% correct). These show clearly that discrimination thresholds for all animals decrease with increasing target bandwidth, and that individual differences are consistent across bandwidth. Figure 3F shows the threshold for localization depends on the signal location (averaged across ferrets). This clearly shows that thresholds for pure tones at 0° are higher than compared with either pure tones at lateral locations (confirmed with an unpaired *t* test, *p* < .05).

## Discussion

Studies of absolute sound localization in noise have shown that, in humans, performance is relatively unaffected until 0–6 dB SNR for broadband signals (Good & Gilkey, 1996; Lorenzi et al., 1999b). Minimal audible angles are also robust to noise, showing no degradation in acuity at −5 to 5 dB SNR for pure tone signals (Jacobsen, 1976). These and other studies suggest that although fairly robust in noise, localization performance begins to decay at SNRs for which signals should still be readily detected (Cherry, 1953; Egan & Benson, 1966; Good & Gilkey, 1996; Jacobsen, 1976; Lorenzi, Gatehouse, & Lever, 1999a). However, it was not always clear how close to signal detection threshold these values were and it is possible that these values in part reflected masking of some components of the signals. Three-location discrimination performance in ferrets declined with SNR. These data are qualitatively consistent with previous findings in humans, though like previous measures of localization performance in ferrets, performance was somewhat worse overall than seen in humans. Pure tone performance was reduced from ∼25 dB SNR (see Figure 3) and although performance for two-octave noise was robust at positive SNRs, more comparable to human performance, it might have been less robust with more signal locations. A further observation from these data is that localization of pure tones in noise is worst for the 0° location, coincident with the masker. This might be expected from the known effects of spatial unmasking reported in the human literature (Hirsh, 1948a). This will be discussed in further detail later in the article.

Increasing the bandwidth of a signal has been shown to improve localization performance both in silence (Boerger, 1965*;* Butler, 1986*;* Terhune, 1974) and in noise (Jacobsen, 1976; Lorenzi et al., 1999a). Not only is our data consistent with this, but the manipulation of signal bandwidth here allows us to infer the source of performance limitations. It is well known that if the overall signal level is fixed, signal detection thresholds increase as the bandwidth of a band-pass signal increases beyond the critical bandwidth of an auditory filter (Spiegel, 1981). Because in this experiment, widening bandwidth improved performance, leading to lower thresholds and steeper psychometric functions, pure tone localization performance is likely to reflect limitations in localization rather than detection.

## Experiment 3: Two-Location (Left/Right) Discrimination in Noise

The previous experiment demonstrated that 3-location discrimination performance depended on spatial cues which were degraded at SNRs where the sounds were still audible. In this experiment, the complexity of the localization aspect of the task was reduced by presenting sounds from only two speakers, at 90° and −90°. Psychometric functions of two-location discrimination in noise were collected using two different methods: limits and constant stimuli. The ability of the ferret to perform a two-location discrimination paradigm was tested and contrasted with existing data on a more conventional yes/no 1I2AFC paradigm.

## Results

**Figure 4 fig4:**
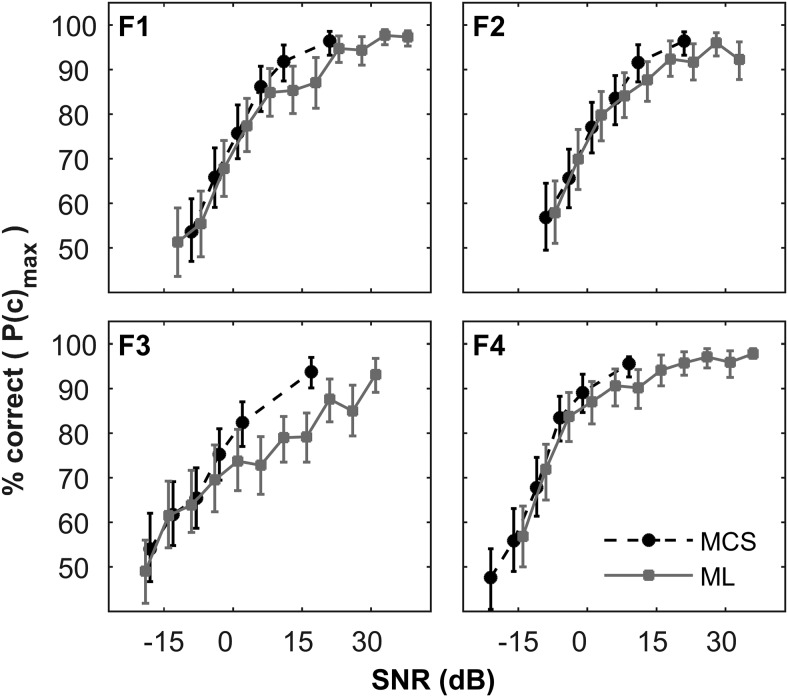
Two-location (left/right) discrimination performance for 10-kHz pure tones in noise as a function of signal-to-noise ratio (SNR) for F1–4, individually. Lines show psychometric functions collected using the method of constant stimuli (MCS) and method of limits (ML; SNR offset slightly for clarity). Error bars indicate 95% confidence intervals.

**Figure 5 fig5:**
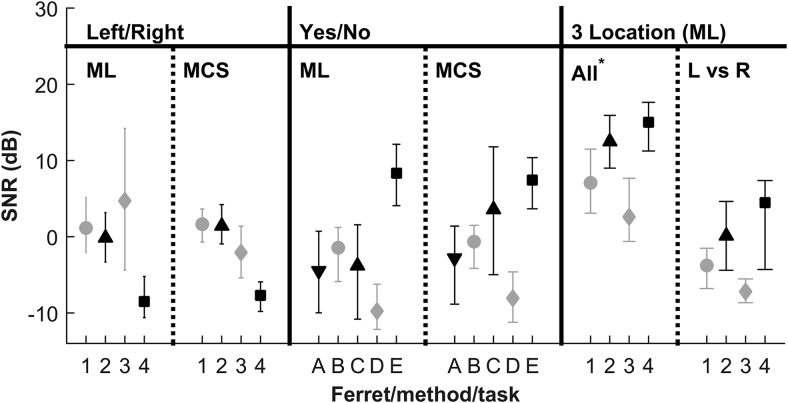
Comparison of individual two-location (left/right) discrimination, detection (yes/no) thresholds, signal-to-noise ratio (SNR) at 75% P(c)_max_, and three-location discrimination in noise. Error bars show 95% confidence intervals. * Three-location thresholds averaged across all locations are drawn from raw % correct scores at 75%. Other data points are thresholds calculated from PC_max_.

Psychometric functions for the two-location discrimination (left/right) task are shown in Figure 4, for each animal individually. Functions collected using the method of limits (black circles with dotted line) and method of constant stimuli (gray squares with solid lines) are shown separately. Here, performance is expressed using the bias-free measure of performance, PC_max_, derived using SDT (see Materials and Method). Qualitatively, in comparison with the pure tone data in Figure 3 all functions are shifted to lower SNRs (cf. horizontal axis values), and in most cases performance remains high (>80%) at positive SNRs. Overall, performance is very similar for the two collection methods. A three-way probit analysis (DeCarlo, 1998; SNR × Method × Ferret) showed that there were significant differences between individual ferrets, χ^2^(18) = 319.9, *p* < .001, but no effect of method, χ^2^(12) = 11.5, *p* = .49. Figure 5 (left) shows the threshold SNRs derived from the left/right discrimination task (where threshold is taken as SNR at PC_max_ = 75%). Consistent with the probit analysis this shows variability between animals, but also good consistency between collection methods for each ferret, with the exception of F4 for the method of limits.[Fig-anchor fig4][Fig-anchor fig5]

To determine whether performance in this task was likely to be limited by either the ability localize or detect the signals, we compared thresholds measured using a signal detection paradigm (yes/no paradigm, Alves-Pinto et al., 2012) with the left/right discrimination paradigm applied here. These data were collected using the same behavioral equipment, though using a different population of animals. Data were processed in an identical manner (see Materials and Method). Figure 5 (right side) also displays the thresholds measured in five ferrets performing a yes/no task. These data were also collected using both method of constant stimuli and method of limits. Overall mean thresholds using these methods were very similar (left/right task: −1.1 dB SNR; the yes/no task: −1.2 dB SNR). A two-way ANOVA with task as a between subjects factor revealed no significant effects, task: *F*(1, 7) = 0, *p* = .98, method: *F*(1, 7) = 0.41, *p* = .54. In Figure 5, the confidence intervals for the thresholds appear to be larger for the yes/no task. However, this difference was not significant, *F*(1, 7) = 0.54 *p* = .48 (two-way between-subjects ANOVA on the bootstrapped confidence intervals). Thus from these data, the two data collection methods appear to yield very similar thresholds.

## Discussion

For convenient visual comparison, thresholds for location discrimination of pure tones in the Experiment 2, averaged across location, are shown in Figure 5 (marked as “all”). Performance on the left/right discrimination task was better than when discriminating pure tones from three locations. Reducing the set of potential sources and increasing source separation improved performance. This again suggests that performance for the three-location discrimination of pure tones was limited by their ability to localize. It is not possible to compare these values quantitatively in this form due to the differences in task and analysis methods: Experiment 2 thresholds are analyzed as raw percent correct because a similar PC_max_ measure is not possible for three locations, and chance performance is different, so we cannot compare it with raw % correct values for Experiment 3, either. Therefore, a more detailed comparison is presented in the Comparisons Across Localization Tasks section.

Two data collection methods were compared for the left/right discrimination task, as well as the yes/no task. For both tasks, both data collection methods produced relatively similar psychometric functions (see Figure 4), and thresholds (see Figure 5) were not significantly different. These conclusions thus extend our previous findings for the yes/no task (Alves-Pinto et al., 2012). The literature broadly supports this result in other species (see Alves-Pinto et al., 2012, for a more detailed discussion).

The left/right discrimination task produced similar thresholds to the yes/no 1I2AFC detection task. This suggests that the left/right task is effectively measuring detection thresholds. The degree to which we can be sure of a quantitative correspondence is necessarily limited the number of subjects (four and five) and the between subject variability (evident in Figure 5). However, another important consideration in comparing these thresholds is whether there were acoustic differences between the tasks. In the yes/no task, signal and masker were collocated, whereas in the left/right discrimination task signals were separated from the masker by 90°. It is possible, therefore, that the SNRs at the ears would be better for the left/right task, producing a spatial unmasking effect. Consistent with this, in Experiment 2, localization was reliably poorer for pure tones from 0°, coincident with the masker, than for lateral locations. However, the available measurements of ferret head-related transfer functions suggest that presenting stimuli from left and right would be expected to either not change the signal level or decrease it by 6 dB (Campbell et al., 2008; Carlile, 1990, and raw data courtesy of Jan Schnupp), compared with signals from directly ahead, at the better ear. Another consideration is whether binaural unmasking might have influenced thresholds. However, binaural masking level differences (BMLDs) are very small at high frequencies (at most, 3 dB) and become smaller in free-field situations (Durlach & Colburn, 1978; Saberi, Dostal, Sadralodabai, Bull, & Perrott, 1991). Because interaural level differences are significant at 10 kHz (>10 dB), the signal is presumably monaural for the left/right task at low SNRs and diotic for the yes/no task. Without any binaural masking release, for the yes/no task, being able to listen for the target with both ears would, in humans, confer an approximately 3-dB advantage (Diercks & Jeffress, 1962). We did not measure transfer functions in these ferrets; therefore, we cannot definitively rule out an influence of directionality on thresholds. Overall, however, there is little reason to think that either lateral or forward stimulus locations had a clear advantage over the other. To determine the correspondence between the tasks to a greater accuracy would probably require a within subject design, which would entail retraining of animals on a different task.

### Comparisons Across Localization Tasks

As the number of sources is reduced and the spacing between them increases, the ability of ferrets to localize narrowband signals in noise improves. This appears to reflect suprathreshold localization limitations, which lead to gradually increasing deficits as SNR is reduced. Experiment 3 showed that thresholds were similar for both left/right discrimination and the yes/no detection task. This suggests that performance in the left/right discrimination task is limited by the ability to hear, rather than the ability to localize. This conclusion is of course contingent on there being little release from masking when a 10-kHz tone is moved from midline to 90°. This is consistent with published data on the directional sensitivity of sound at this frequency. However, it seems inconsistent with observed higher thresholds for localization of tones at 0° in Experiment 2 (Figure 3A, B, and F). It also does not provide a quantitative account of how or why performance at lateral locations appears qualitatively different in Experiments 2 and 3.

### Signal Detection Models

**Figure 6 fig6:**
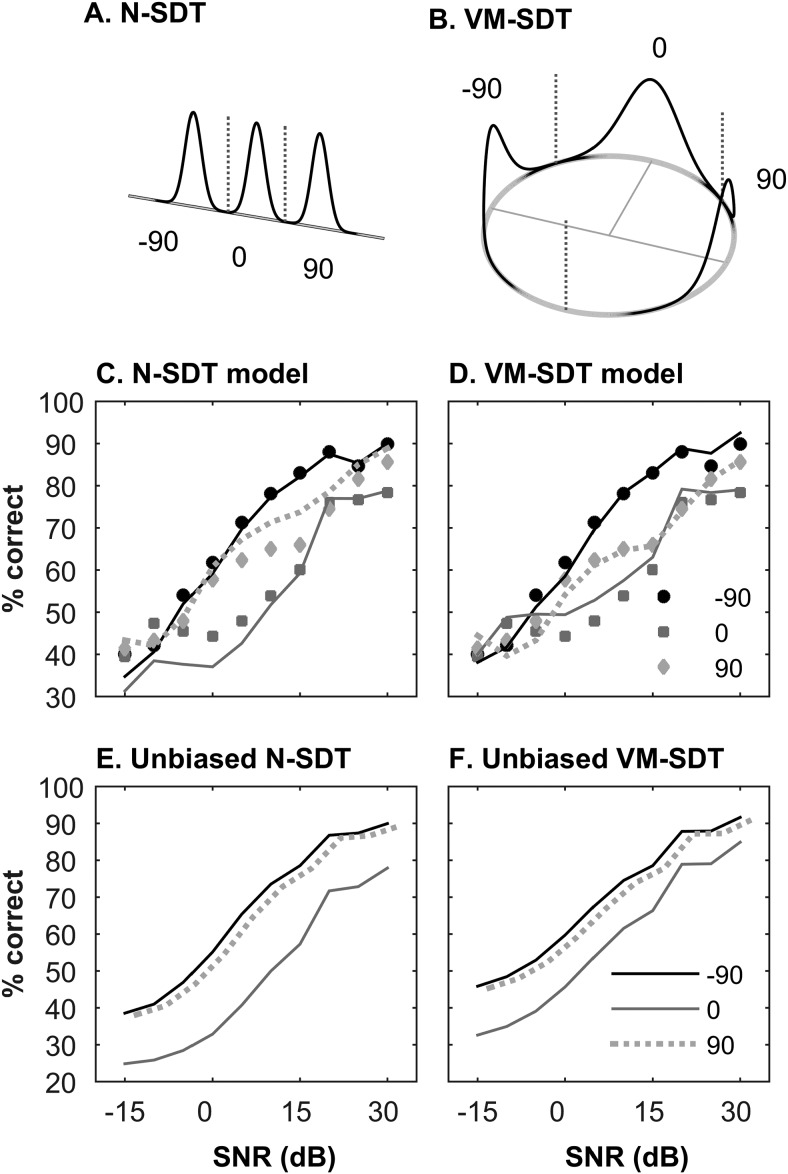
Signal-detection models of Experiment 3. (A) The internal representation for a normal signal detection theory (N-SDT) model with a linear spatial dimension, normally distributed representations of source location and putative ideal discrimination criterion locations. (B) The internal representation of the von Mises signal detection model (VM-SDT) showing a circular spatial dimension, example distributions associated with the three signal locations and ideal criterion locations. (C) The results of fitting the N-SDT model (solid lines) to the data (points) in Experiment 3, averaged across subjects. (D) The results of fitting the VM-SDT model. (E–F) The N-SDT and VM-SDT model performance when the decision criterion are fixed as optimal and unbiased (90° lines are displaced slightly horizontally for clarity).

To gain insight into the relationship between Experiments 2 and 3, we fitted simple SDT models to the data. We used two kinds of model. One was a classical normal SDT (N-SDT) model based on the assumption that there is a linear internal representation of acoustic space, and that sound sources are represented as normally distributed random variables (Figure 6A). The internal space was in units of azimuthal angle, and the variance of the distributions determined how discriminable different spatial locations were. Decisions about sound location are made by comparing the random variables to decision criterion (as shown in Figure 6A). Thus, for a given source location, the confusion matrix can be calculated by calculating the probability of answer *a*_*i*_ given the speaker *s*_*j*_ at location ω_*j*_ by integrating the normal probability density function between the criterion limits, according to the following equation:

$$P\left(a_{i}|s_{j}\right)=\;\frac{1}{\sigma \sqrt{2\pi }}\int_{k_{i-1}}^{k_{i}}e^{\frac{-{\left(\theta -\omega_{j}\right)}^{2}}{2\sigma }}d\theta ,$$ 3

[Fig-anchor fig6] where *k*_*i–1*_ and *k*_*i*_ come from the ordered set {-∞, *k*_*1*_, *k*_*2*_, ∞}. Criterion locations *k*_*1*,_ *k*_*2*_ and the distribution width σ and are free parameters in the model. The distribution width is fixed for all spatial locations at a single SNR, in other words spatial representations were equally accurate at all source locations. The speaker locations are fixed at {–π/2, 0, π/2}. The model is fitted to the full confusion matrix, independently at each SNR.

The N-SDT model does not take account of the nature of azimuthal source location, which is circular. Therefore we also fitted the data to SDT models based on the von Mises distribution (VM-SDT), which can be considered as a circular close equivalent to the normal distribution (Figure 6B). Model fitting and constraints were as for the N-SDT model, except that the circular nature of the representation necessitates an additional decision criterion, to divide the rear internal space (Figure 6B). The probability of answer *a*_*i*_ given a sound from speaker *s*_*j*_ is specified by

$$P\left(a_{i}|s_{j}\right)=\int_{k_{i-1}}^{k_{i}}\frac{e^{k\cos(\theta -\omega_{j})}}{2\pi I_{0}(\kappa )}d\theta \;,$$ 4

where criterion *k*_*i*–1_ and *k*_*i*_ belonged to the ordered set of free parameters {*k*_0_, *k*_1_, *k*_2_}, shape parameter κ, which defined the narrowness of the distributions, was a single free parameter for all speaker locations, and speaker locations ω_*j*_ were fixed at {–π/2, 0, π/2}. All parameters and variables are specified in radians. All simulations and model fitting was performed in MATLAB, and the von Mises calculation was performed using the circular statistics toolbox (Berens, 2009). Models outputted simulated confusion matrices calculated from the above equations, which were then subject to the same analyses as the data.

### Modeling Results

Figure 6C and 6D show the results of fitting the two SDT models to the three-location data. The VM-SDT model is a better fit to the data (χ^2^ goodness-of-fit: 30.0) than the N-SDT model (50.7). However, both models are able to predict the lower % correct values for signals at 0°. Neither is this purely a consequence of the decision criterion to fit the data. Fixing the criterion equidistant between distributions, which is optimal in the sense of maximizing overall percent correct, much like a PC_max_ calculation in SDT, still results in poorer model performance at 0° (as shown Figure 6E–F). This arises because for signals from straight ahead, there are two other potential source locations 90° away. Thus, in both models, the proximity of relative source locations leads to poorer performance at 0°. This may explain how thresholds for the 0° location can be higher than lateral locations in Experiment 2, even though there is little spatial masking release, and while thresholds for left/right discrimination in Experiment 3 can be similar to detection thresholds in the yes/no task.

While models appear adequate to reproduce qualitatively the main features of the data, note that the two models would differ fundamentally in their predictions for three equally spaced sources (120° apart): The N-SDT model would still predict worse performance for the 0° location, whereas the VM-SDT will predict that performance will be equal across all source locations (not shown) because they will be equidistant in the internal representation.

### Comparing Discrimination of Lateral Locations

Next we will address the question of how the results from Experiments 2 and 3 might be related. For lateral locations, the stimuli are identical. However, it is not possible to compare percent correct values across the two experiments, because chance performance is different (33% vs. 50% correct). Therefore we compute *d*′ between lateral locations in both experiments as per the Data Analysis section (Macmillan & Creelman, 2005) according to Equation 2:

$$d\prime =\frac{z(R_{L}|S_{L})-Z(R_{L}|S_{R})}{2},$$ 5

**Figure 7 fig7:**
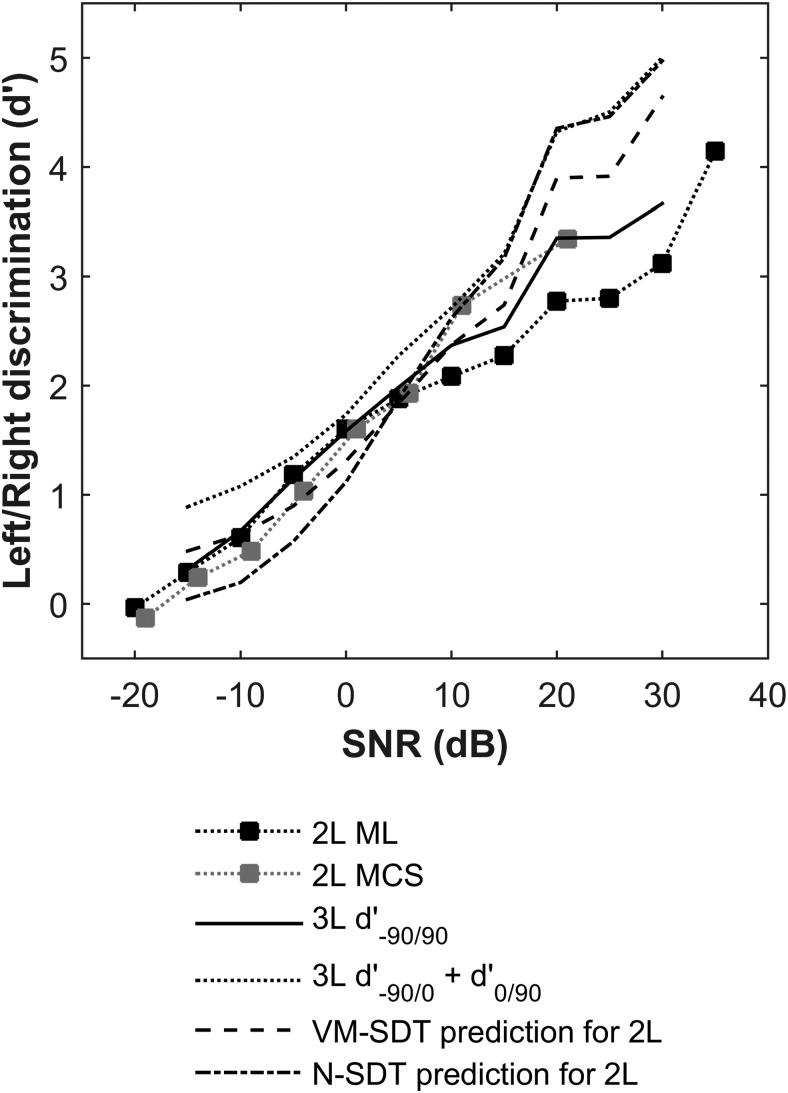
Comparisons of left/right discrimination drawn from Experiments 2 and 3, and from the models fitted to Experiment 2.

Figure 7 shows discrimination performance between pure tones from ±90° calculated from a variety of different measurements. *d*′ values calculated from Experiments 2 for these locations and from Experiment 3, collected using the method of limits or the method of constant stimuli, are in good agreement at 10 dB SNR and lower. Data and predictions tend to diverge at higher SNRs, and paired *t* tests conducted between the two datasets from Experiment 3 left/right discrimination performance calculated from Experiment 2 revealed that differences were significant (*p* < .05 uncorrected). Figure 5 shows the thresholds for discrimination of −90°/90° locations in Experiment 2 calculated in the same way as for Experiment 3 (i.e., from PC_max_ using Equation 2). Although the correspondence is not perfect, we would argue that these analyses suggest that the differences in performance between Experiments 2 and 3 are largely down to the change in the number and proximity of source locations.[Fig-anchor fig7]

It is also possible to use the models fitted to the data from Experiment 2 to predict performance in Experiment 3. Figure 7 shows the results of running the models using the distribution widths (N-SDT: σ; VM-SDT: *k*) fitted to the data in Experiment 2, but considering only two source locations at +/−90°. Here, criteria are assumed to be placed optimally and equidistant between sources (i.e., 0° and −180°; note that criterion is actually irrelevant for the *d*′ calculation). The VM-SDT and N-SDT models are able to predict left/right discrimination at a wide range of SNRs (paired *t* tests of the model *d*′s with those of Experiment 3 show no significant differences, *p* ≥ .3).

SDT also allows a further, analytical, test of the validity of the assumption made by this model. If the internal space is linear, then *d*′ should sum linearly across it (Macmillan & Creelman, 2005). In other words, SDT predicts that *d*′_–90°/0°_ + *d*′_0°/+90°_ = *d*′_–90°/+90°_. Figure 6 also shows *d*′_–90°/0°_ + *d*′_0°/+90°_, made directly from the data. This prediction of *d*′_–90°/+90°_ is again surprisingly good, although qualitative rather than quantitative (paired *t* tests with Experiment 3, *p* < .05).

### Discussion

This further analysis and modeling of the data appear to allow a reasonably coherent interpretation of the combined results of Experiments 2 and 3. Importantly, SDT analysis of the ±90° locations shows they are discriminated similarly (though not identically) in both experiments. Thus the spatial perception of lateral locations is consistent across experiments. This is further supported by the prediction of the results of Experiment 3 based the VM-SDT model fits to Experiment 2, which shows that a single model of spatial perception can qualitatively, at least, reconcile the results of Experiments 2 and 3. It is not unexpected that a sensory representation would be consistent across tasks, but it was not a forgone conclusion that such a simple model was adequate to show this.

Both SDT models assumed that the spatial accuracy with which each source was represented was identical at a given SNR. Effectively therefore it assumed there was no spatial difference in masking across the three source locations. Nevertheless both models were able to account qualitatively for the poorer localization performance at 0°. This suggests that the data are consistent with a representation where the spatial resolution is relatively similar at 0° and 90°. It is unlikely that such a simple model would be able to account for a larger amount of localization data. For example, increased gain for signals coming from intermediate locations (e.g., 45°) in the frontal field would result in a larger spatial masking release. In addition, it is well established that localization is most accurate for sounds from straight ahead (Hafter, De Maio, & Hellman, 1975; Mills, 1958). This is evident in Figure 2A which, consistent with previous reports in ferrets (Nodal et al., 2008), shows that as the duration of a noise burst is reduced, the performance drop is more marked for sounds coming from behind. This latter result implies that even in silence the width of spatial representations would depend on location, being broader for sounds from behind.

Finally, although the VM-SDT model was quantitatively superior to the N-SDT model, qualitatively, the N-SDT models displayed many of the same properties. It is not surprising that the VM-SDT may provide a more accurate model of sound-localization than a conventional SDT model, which cannot account for the fact that as sounds cross the frontal plane they begin to get closer again. Such a model would certainly not be suitable for sounds coming from front and behind. However, in these experiments the difference was a quantitative rather than gross qualitative one.

## General Discussion

These data reveal some of the perceptual constraints on the localization of high-frequency sounds in ferrets. Ferrets have been used extensively to study sound localization (e.g., Bajo, Nodal, Moore, & King, 2010; Kacelnik et al., 2006; Kavanagh & Kelly, 1987, 1992; Keating, Dahmen, & King, 2015; Kelly & Kavanagh, 1994; King et al., 2011; Leach, Nodal, Cordery, King, & Bajo, 2013; Nodal, Bajo, & King, 2012; Nodal et al., 2010; Parsons, Lanyon, Schnupp, & King, 1999). In quiet, minimal audible angles for broadband noise are of the order of 10–30° for the stimulus durations used here (500 ms; Parsons et al., 1999), and interaural-level-difference (ILD) thresholds are of the order of 1–3 dB (considering variations in sound level, duration; Keating & King, 2013). For narrower bandwidth signals localization performance decays markedly at high frequencies (<60% correct for 1-s long, one sixth octave noise bands centered at 15 kHz; Kacelnik, et al., 2006), consistent with the effect of bandwidth observed for humans (Boerger, 1965; Butler, 1986; Terhune, 1974).

In all our experiments, the ferrets were able to localize signals presented to them at high SNRs. However, when separated by 90°, 10-kHz tones became progressively more difficult to localize with decreasing SNRs. While we do not know of any other data looking at sound localization in noise in animals, it is nevertheless consistent with previous data in as much as, as well as humans, other species are also able to localize pure tones in quiet, but performance is usually worse than for broadband sounds (e.g., starlings: Feinkohl, Borzeszkowski, & Klump, 2013; gerbils: Heffner & Heffner, 1988; chinchillas: Heffner, Heffner, Kearns, Vogel, & Koay, 1994; cats: Martin & Webster, 1987). It is clear in Experiment 2 that at positive SNRs at least this was attributable to a problem localizing rather than hearing.

The constraints seen here for 10-kHz tones may well be different for other frequencies or signals. For instance, the BMLD, where the phase disparity of the masker and signal are different, can lead to reductions in threshold (Blodgett, Jeffress, & Taylor, 1958; Hirsh, 1948b; Jeffress, Blodgett, & Deatherage, 1962; Licklider, 1948). At high frequencies, interaural phase becomes ambiguous, and in the ferret at least phase locking drops off above 1 kHz and is low by 3 kHz (Sumner & Palmer, 2012) and so could not have been used in this task. However, at low frequencies in humans, large BMLDs can be found, for example, 15 dB at 500 Hz; above 1,500 Hz, the BMLD is only 3 dB (Durlach & Colburn, 1978). Binaural unmasking has been demonstrated in ferrets (Hine et al., 1994) in the free field, and, therefore, it is likely that for low frequency signals, ferrets would be able to perform this task by capitalizing on interaural phase cues. Therefore, one might expect that BMLD confers an advantage at low frequencies resulting in lower SNR thresholds relative to diotic stimuli such as used in the yes/no task. Likewise, one might expect that three-location discrimination might be more robust in noise for low-frequency tones.

Another effect that may influence thresholds is spatial unmasking. In humans, sounds presented with equal amplitude and at an equal distance from the head are easiest to detect when presented from ∼45° azimuth (Saberi et al., 1991; Sabin, Macpherson, & Middlebrooks, 2005). In ferrets, there are a number of noticeable differences that occur, for example, differences in size and shape of the head and pinna. For the purposes of this discussion, we are only interested in the difference in the signal level when presented ±90° and when compared to 0°. This does depend somewhat on frequency. However, although pinna related gain does vary with frequency, the difference in gain between 0° and 90° (for the better ear) for a given frequency varies much less because, like in humans, maximum gain occurs at intermediate angles (∼45°; Campbell et al., 2008; Carlile, 1990; and raw data from Jan Schnupp). Thus, monaural cues for spatial masking release are not expected to be very large at most frequencies.

A novel aspect of this study was the application of signal detection models to help explain our results. These models confirmed that spatial masking was not necessary to explain the data. Indeed the models provided a framework to explain both Experiments 2 and 3, and suggested an interplay between localization ability, detection, and the contingencies of the task (number and location of possible sources). We were unable to find any other applications of von Mises distributions to sound localization in the literature, although they have been used to model the visual perception of bar orientation (van den Berg, 2012). Here, we found the approach highly adequate. However, we expect that such models are only likely to be useful in restricted situations. The VM-SDT model assumes a unidimensional representation of azimuthal space and it is difficult to envisage how such a model could account for all aspects auditory-spatial perception such as front–back confusions or the integration of level and timing cues.

We expected that the approach-to-target task might actually confer some advantages over the yes/no task. The classical literature describes how learning depends on task implies that the left/right discrimination task should be learned more rapidly than the yes/no task (Harrison, 1992). Our experiments were not configured ideally for comparing learning rates, because the left/right discrimination task was preceded by a general sound localization task and then a three location discrimination task. However, some other task configurations can prevent learning of the task to a high standard at all (Burdick, 1979; Harrison, 1992; Neill & Harrison, 1987). While this is clearly not a problem with the yes/no detection task, it is possible that the choice of task might also impact on the reliability of measurements in “trained” animals and of thresholds. However, this effect in our data at least appeared to be subtle and did not reach significance.

Furthermore, a theoretical advantage of the left/right over the yes/no task relates to the differing requirements for setting and maintaining a decision criterion. In any forced choice task there is the possibility for response-bias. In the yes/no task, this is determined by a decision criterion value, against which a noisy internal variable is compared. The presence or absence of a signal and the sound level is directly related to this internal variable. Indeed, to maximize performance this criterion must be altered dependent on the set of stimuli presented and held in memory (e.g., one sound level in a session using the method of limits, or many sound levels for the method of constant stimuli), and the adjustment of criterion can be observed from trial-to-trial (Mill, Alves-Pinto, & Sumner, 2014). The need for criterion adjustment is true for go/no-go tasks (see MacMillan & Creelman, 2005). In the left/right task, the required decision is not whether a sound occurred but which side the sound came from. There is an ideal criterion but it relates to a comparison across the two ears or a judgment about perceived location. It does not need to vary with the set of stimuli presented, and may have lower demands on memory. It seems possible that this theoretical difference in demands on criterion setting between the yes/no and left/right tasks could influence in the variability of measurements. However, again we note the effect is subtle in our data.

In summary, we examined location discrimination of band-limited high-frequency signals in noise behaviorally in ferrets. Dependant on the stimulus configuration, performance was limited by either the ability to localize or to detect the signals. Further analysis and modeling of Experiments 2 and 3 showed that although the results in Experiments 2 and 3 were quite different, they were nevertheless consistent with the same underlying sensory representation. Models were also able to show that the differences in performance at different locations in Experiment 2 were likely attributable to task differences rather than physical differences in SNR with location. Discriminating signals from two locations, on the left and the right, produced thresholds that were similar to thresholds collected using a more conventional yes/no detection task. At the least, this difference was smaller than the variability between subjects. This suggests that both tasks were measuring the same perceptual limits of signal detection, and that there was minimal interaction of the nature of the task in these measurements. An important practical implication of this is that an approach-to-target task can, with care, be used to measure detection thresholds. Finally, we have demonstrated it is possible to train ferrets on one task (approach-to-target) to both localization and detection thresholds, removing the need to train them on two separate tasks.
